# IL-33 and its decoy sST2 in patients with Alzheimer’s disease and mild cognitive impairment

**DOI:** 10.1186/s12974-020-01806-4

**Published:** 2020-06-06

**Authors:** Marina Saresella, Ivana Marventano, Federica Piancone, Francesca La Rosa, Daniela Galimberti, Chiara Fenoglio, Elio Scarpini, Mario Clerici

**Affiliations:** 1 IRCCS Fondazione Don Carlo Gnocchi, Laboratory of Molecular Medicine and Biotechnology, Via Capecelatro, 66, 20148 Milan, Italy; 2 Department of Biomedical, Surgical and Dental Sciences, University of Milan, Milan, Italy; 3 Department of Pathophysiology and Transplantation, University of Milan, Milan, Italy; 4 Centro Dino Ferrari, Milan, Italy; 5 Fondazione IRCCS Ca’ Granda, Ospedale Policlinico, Milan, Italy

**Keywords:** Alzheimer’s disease, Inflammation, Interleukin 33, sST2 decoy receptor

## Abstract

**Background:**

Interleukin-33 is a cytokine endowed with pro- and anti-inflammatory properties that plays a still poorly defined role in the pathogenesis of a number of central nervous system (CNS) conditions including Alzheimer’s disease (AD). We analyzed this cytokine and its decoy receptor sST2 in Alzheimer’s disease (AD) and mild cognitive impairment (MCI).

**Method:**

IL-33 and sST2 were analyzed in serum and CSF of AD and MCI patients, comparing the results to those obtained in age-matched healthy controls (HC). Because of the ambiguous role of IL-33 in inflammation, the concentration of both inflammatory (IL-1β and IL-6) and anti-inflammatory (IL-10) cytokines was analyzed as well in serum and cerebrospinal fluid (CSF) of the same individuals. Finally, the effect of IL-33 on in vitro Aβ_42_-stimulated monocytes of AD, MCI, and HC individuals was examined.

**Results:**

As compared to HC, (1) IL-33 was significantly decreased in serum and CSF of AD and MCI, (2) sST2 was increased in serum of AD and MCI but was undetectable in CSF, (3) serum and CSF IL-1β concentration was significantly increased and that of IL-10 was reduced in AD and MCI, whereas no differences were observed in IL-6. In vitro addition of IL-33 to LPS+Aβ _42_-stimulated monocytes downregulated IL-1β generation in MCI and HC, but not in AD, and stimulated IL-10 production in HC alone. IL-33 addition also resulted in a significant reduction of NF-kB nuclear translocation in LPS+Aβ_42_-stimulated monocytes of HC alone.

**Conclusions:**

These data support the hypothesis that IL-33 plays a complex anti-inflammatory role that is lost in AD- and MCI-associated neuroinflammation; results herein also suggest a possible use of IL-33 as a novel therapeutic approach in AD and MCI.

## Background

Interleukin-33 (IL-33) is a dual function cytokine produced by endothelial and epithelial cells as well as fibroblast, macrophages, adipocytes, smooth muscle, and brain cells [1]. This cytokine is released as a full-length active protein that can be inactivated by caspase-1, caspase-3, and caspase-7-mediated cleavage [2, 3] or can processed by different proteases into shorter forms characterized by diverse biological activities [4–6]. The regulation of IL-33 biological activity is complex as it is the result of the interplay between the extra- and intracellular forms of this cytokine and its ability to bind two different isoforms of ST2, its cognate receptor. Binding of extracellular IL-33 to ST2 on leukocytes, astrocytes, and oligodendrocytes [7] mediates the biological effects of this cytokine [8]. On the other hand, when expressed intracellularly, IL-33 binds the p65 subunit of NF-kB [9]; the resulting IL-33/NF-kB p65 complex interferes with NF-kB-dependent transcription by impeding p65-mediated transactivation. This causes a negative modulation of NF-kB activity, with a dampening effect on inflammation. An additional actor that modulates the biological activity of IL-33 is the soluble form of ST2 (sST2), a decoy receptor. As is usually the case with decoy receptors, IL-33 binding to sST2 limits its biological activity [10].

Th2 helper cells and mast cells express ST2 and respond to IL-33, and type 2 innate lymphoid cells (ILC2) are considered to be the signature IL-33-responsive cells [11]. Thus, IL-33 targets ILC2 to produce IL-5 and IL-13, resulting in the recruitment of eosinophils, the activation of DC, and Th2 differentiation [12–16]. A growing body of evidence suggests that IL-33 also controls the accumulation and effector function of regulatory T cell (Treg), either directly or indirectly via ILC2 activation and macrophage polarization [17–19]. Thus, IL-33 causes microglial and macrophage polarization to an anti-inflammatory type-2 (M2) phenotype through the IL-33/ST2 signaling pathway; this results in IL-10 generation and reduces IL-1β and IL-6 production [20–22].

IL-33 plays a yet poorly understood role in the pathogenesis of Alzheimer’s disease (AD), a condition where deposition of extracellular amyloid beta (Aβ) plaques in the brain and neuronal cell death accompany neuroinflammation. In AD patients, the concentration of proinflammatory cytokines, including interleukin IL-1β is increased, possibly as a result of the activation of the NLRP3 inflammasome, and immune regulatory mechanisms, including those mediated by Tregs and PDL-1, are impaired [23–27]. A more subtle form of neuroinflammation is also present in mild cognitive impairment (MCI), a subjective and objective decline in cognitive performance that is greater than expected for an individual’s age and education level, but does not meet criteria for the diagnosis of AD [28]. Elderly MCI patients are at high risk for developing AD; this situation thus represents a borderline condition between normal aging and AD [29].

Correlates of MCI conversion to AD are still poorly defined, even if neuroinflammation is strongly suspected to play a role in this process [29–33]. Recent observations suggest a beneficial role of IL-33 in AD-associated neuroinflammation. To summarize, (1) ex vivo results in cellular models of AD indicated that over expression of IL-33 decreases Aβ secretion and (2) autoptic data indicate that IL-33 is significantly reduced in brains of AD patients. Notably, in the APP/PS1 animal model of AD, IL-33 administration was shown to restore the phagolysosomal activity of the microglia, thus enhancing amyloid β (Aβ) clearance, and to polarize monocytes to an anti-inflammatory phenotype. This resulted in a significant amelioration of AD symptoms [34], suggesting the possibility that IL-33 is a neuroprotective cytokine in AD [35]. This hypothesis nevertheless is not supported by other results suggesting that IL-33 is present in high concentrations in the neuropathological lesions of AD brain and can exacerbate AD-associated neuroinflammation [36]. In the attempt to clarify the role of the ST2/IL-33 axis in AD, we analyzed these proteins in AD, MCI, and healthy controls (HC) individuals and explored the effect of IL-33 supplementation in an in vitro system of Aβ-stimulated monocytes.

## Materials and methods

### Patients and controls

Ninety elderly Italian individuals were enrolled in the study by the Neurology Department of the IRCCS Ca’ Granda Ospedale Maggiore Policlinico, Italy. Thirty patients had a diagnosis of AD, 30 patients had a diagnosis of mild cognitive impairment (MCI), and 30 individuals were age and sex matched healthy controls (HC). The clinical diagnosis of AD was performed according to NINCDS-ADRDA work group criteria [28] and further revisions [37], the clinical diagnosis of MCI fulfilled Petersen’s operational criteria [29]. Neuropsychological evaluation was performed with a Mini-Mental State Examination (MMSE) [38] and Clinical Dementia Rating Scale (CDR) [39].

All patients underwent a clinical interview, neurological and neuropsychological examination, routine blood tests, brain MRI, and lumbar puncture (LP). The mean age of AD patients (13 males and 17 females) was 74.3 years (age range 54–88 years) and that of MCI patients (8 males and 22 females) was 75.3 years (age range 63–84 years). All the AD patients were enrolled in a Multidimensional Stimulation Rehabilitation program designed for this pathology. Finally, the thirty age- and sex-matched elderly subjects (HC) who were included in the study were selected according to the SENIEUR protocol for immuno-gerontological studies of European Community’s Control Action Programme on Aging [40]; their MMSE score was > 28.

The study was approved by the Ethics Committee of Don Gnocchi Foundation and informed written consent was obtained from all the included subjects before study initiation.

### Serum and CSF

Serum was collected in vacutainer tubes containing serum separator (Becton Dickinson and Co., Rutherford, NJ, USA) and were centrifuged at 3,000 rpm for 10 min to separate sera; CSF samples were collected by lumbar puncture. Serum and CSF were used immediately or stored at − 80 °C.

### Blood sample collection and cell separation

Whole blood of a subset of individuals (5 AD, 5 MCI, and 5 HC) was collected in vacutainer tubes containing ethylenediaminetetraacetic acid (EDTA) (Becton Dickinson). Peripheral blood mononuclear cells (PBMC) were separated on lympholyte separation medium (Cedarlane, Hornby, Ontario, CA, USA) and washed twice in PBS at 1500 rpm for 10 min; viable leukocytes were determined using a TC20 Automated Cell Counter (Biorad, Hercules, CA, USA).

### Cell cultures

PBMC (1 × 10^6^/ml) were cultured in RPMI 1640 supplemented with 10% human serum, 2 mM l-glutamine, and 1% penicillin (Invitrogen Ltd, Paisley, UK) and incubated at 37 °C in a humidified 5% CO_2_ atmosphere for 2 h in a 12-well plate for monocyte adhesion. After 2 h, non-adhering PBMC were harvested and discarded and monocytes grown on plate were either culture in medium alone (unstimulated) or were or primed with 2 μg/ml lipopolysaccharide (LPS) **for 2 h (Sigma-Aldrich, St. Louis, MO, USA)** before stimulation with 10 μg/ml of 1-42 amyloid-beta peptide (Aβ_42_) **(Sigma-Aldrich)** in the absence/presence of 10 ng/ml of Human Recombinant IL-33 (Biolegend, San Diego, CA, USA) for 24 h at 37 °C in a humidified 5% CO_2_ atmosphere. After 24 h, supernatants were collected and stored at − 20 °C; adhering cells (monocytes) were collected and prepared for FlowSight analysis.

### Enzyme-linked immunosorbent assay (ELISA)

IL-33 (catalog number D3300B, sensitivity 1.51 pg/mL, assay range 3.1–200 pg/ml, minimum detectable dose (MDD) ranged from 0.069 to 1.51 pg/mL), sST2 (catalog number DST200, sensitivity 13.5 pg/mL, assay range 31.3–2000 pg/mL, MDD ranged from 2.45 to 13.5 pg/mL), IL-1β (catalog number DLB50, sensitivity 1 pg/mL, assay range 3.9–250 pg/ml, MDD less than 1 pg/mL), IL-6 (catalog number D6050, sensitivity 0.70 pg/mL, assay range 3.1–300 pg/ml, MDD less than 0.70 pg/mL), and IL-10 (catalog number D1000B, sensitivity 3.90 pg/mL, assay range 7.8–500 pg/ml, MDD less than 3.90 pg/mL) concentration was analyzed in serum and CSF by commercially-available ELISA according to the manufacturer’s recommendations (Quantikine Immunoassay; R&D Systems, Minneapolis, MN, USA, or Thermo Fisher Scientific, Waltham, MA, USA). Serum and CSF samples were not diluted prior to being utilized. A plate reader (Sunrise, Tecan, Mannedorf, Switzerland) was used and optical densities (OD) were determined at 450/620 nm. All samples were performed in duplicates. The same methods were used to measure IL-1β, IL-6, and IL-10 in unstimulated and in LPS-primed and Aβ_42_-stimulated PBMC supernatants in the presence/absence of recombinant IL-33 (see above).

### Western blot

A Qubit Protein Assay Kit was used for protein extraction; total protein amount was measured using a Qubit 3.0 Fluorometer (Thermo Fisher Scientific). Equal amounts of protein (15 μg) from each sample, or 2 ng of artificial truncated IL-33 (amino acids 112–270, 20 kDa) (Recombinant Human IL33 carrier-free, Biolegend, San Diego, CA, USA) used as positive control to confirm that the bands correspond to IL-33 full length (amino acids 1–270, 34–30 kDa), to the cleaved inactive forms (amino acids 1–178, 22–20 kDa; amino acids 179–270, 13–12 kDa) [2, 3] or, finally, to the cleaved active forms (amino acids 95–270, amino acids 99–270, and amino acids 109–270; 19–15 kDa) [4], were separated by 10% SDS-PAGE and transferred to PVDF membranes (Genscript). A pre-stained marker, broad range 11–190 KDa (Cell signaling, Danvers, MA, USA), was also used, PVDF membrane were treated by ONE-HOUR Western detection kit (Genscript) and proteins were visualized using a Chromosensor TMB substrate (Genscript). After protein transfer, PVDF membranes were incubated with a pretreatment solution for 5 min RT and then with the WB solution and an α-human IL-33 Ab (Nessy-1) (Abcam, Cambridge, UK) for 40 min. After three washes, membranes were developed with a TMB substrate.

### Cell culture and NFkB/7AAD intracellular staining

Monocytes that were either unstimulated or LPS-primed and A**β**_42_-stimulated in the presence/absence of 10 ng/ml of recombinant IL-33 were analyzed to evaluate nuclear translocation of NF-kB; the Amnis® NF-kB Translocation Kit was used according to the manufacturer’s recommendations (Merck KGaA, Darmstadt, Germany). Briefly, cells were fixed, permeabilized, and stained with Anti-Hu NF-κB (p50) Alexa Fluor® 488 for 30 min RT. After incubation, monocytes were washed and fixed and 10 μl of 7AAD were added.

### FlowSight analysis

The FlowSight (Amnis Corporation, Seattle, WA, USA) is equipped with two lasers operating at 488 and 642 nm, two camera, and twelve standard detection channels. It simultaneously produces side scatter (darkfield) images, one or two transmitted light (brightfield) images, and up to ten channels of fluorescence imagery of every cell. FlowSight acquires 2000 cells/s and operates with a 1-μm pixel size (× ~ 20 magnification) allowing visualization of fluorescence from the membrane, cytoplasm, or nucleus. The IDEAS image analysis software allows quantification of cellular morphology and fluorescence at different cellular localizations by defining specific cellular regions (masks) and mathematical expressions that uses image pixel data or masks (feature) by different wizards. Analysis of NF-kB translocation was performed by Nuclear Localization Wizard using the Similarity Feature. Briefly, nuclear translocation has occurred if the NF-κB and nuclear fluorescence signals overlap with similar shapes. The Bright Detail Similarity is designed specifically to compare the small bright image detail of two images and can be used to quantify the co-localization of two probes (NF-kB and 7AAD) in a defined region. The similarity score is the log transformed Pearson’s correlation coefficient and it is a measure of the degree to which two images are linearly correlated within a masked region and is calculated on a double-positive region (NF-kB+7AAD+).

### Statistical analysis

Quantitative data were not normally distributed (Shapiro-Wilk test) and were summarized as median and interquartile range (IQR) (25° and 75° percentile). Comparisons between groups were performed used a Kruskal-Wallis ANOVA for each variable. Comparisons among the different groups were made using a 2-tailed Mann-Whitney *U* test performed for independent samples. Data analysis was performed using the MedCalc statistical package (MedCalc Software bvba, Mariakerke, Belgium).

## Results

### IL-33 isoforms

**Fig. 1:**
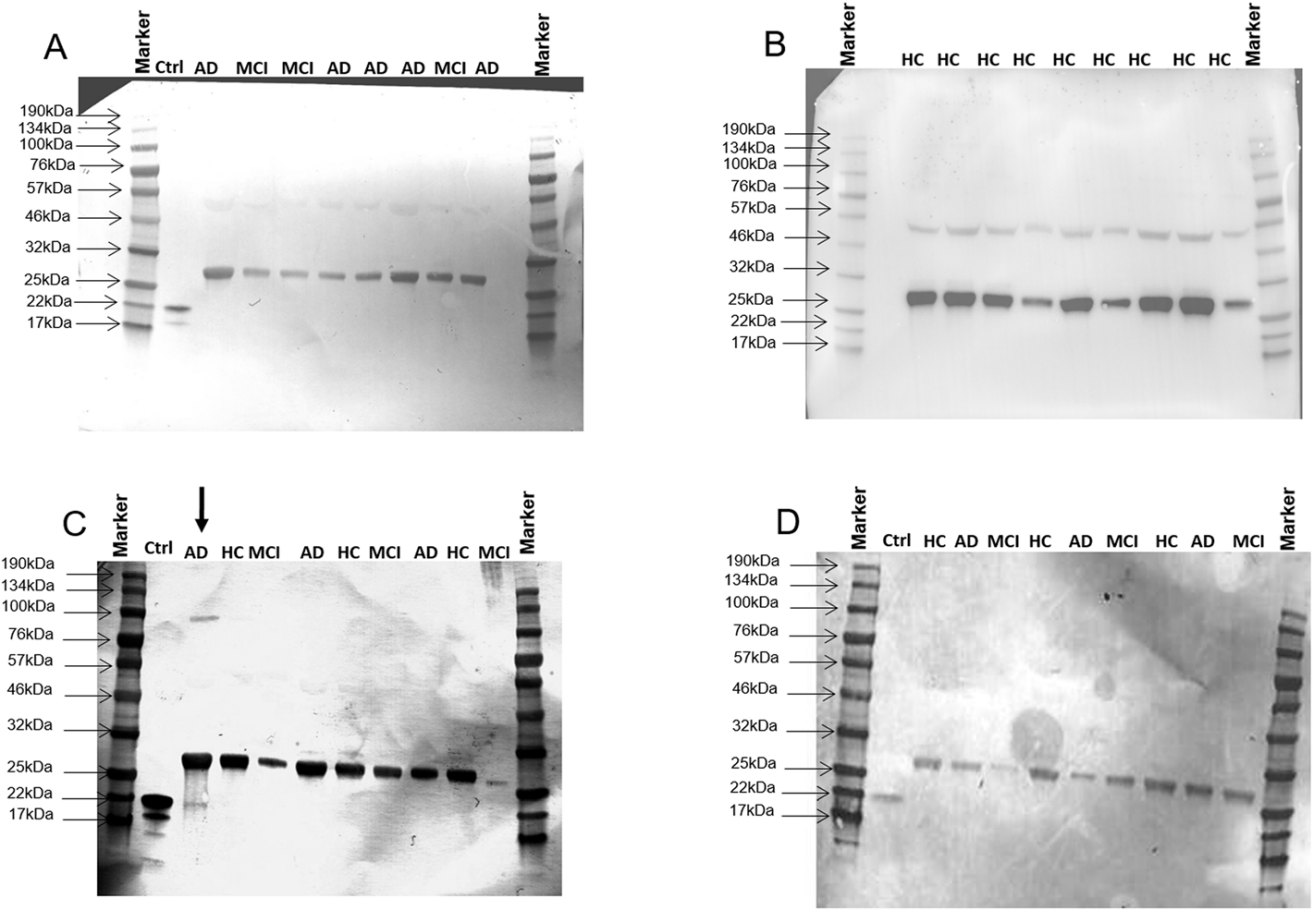
IL-33 isoforms. Interleukin-33 in serum (**a**–**c**) and CSF (**d**) samples of representative AD, MCI, and HC individuals. A 30-kDa band corresponding to the full-length IL-33 protein was detected in all cases. An additional 20-kDa band was also present in two cases. Human recombinant artificial truncated IL-33 (amino acids 112–270, 20 kDa) was used as a positive control (Ctrl); a pre-stained marker was also used. Broad range 11–190 kDa

Analysis of the different isoforms of IL-33 (full-length; cleaved inactive; cleaved active) was performed by Western blotting in serum and CSF of AD, MCI and HC individuals. Results showed that the full-length IL-33 protein (30–kDa band) was present in all analyzed serum and CSF samples. Notably, in two cases an additional 20-kDa band, corresponding to the cleaved (c), inactive form of IL-33 [3] was observed. These results are shown in Fig. 1.

### IL-33 and sST2 serum and CSF concentration

**Fig. 2:**
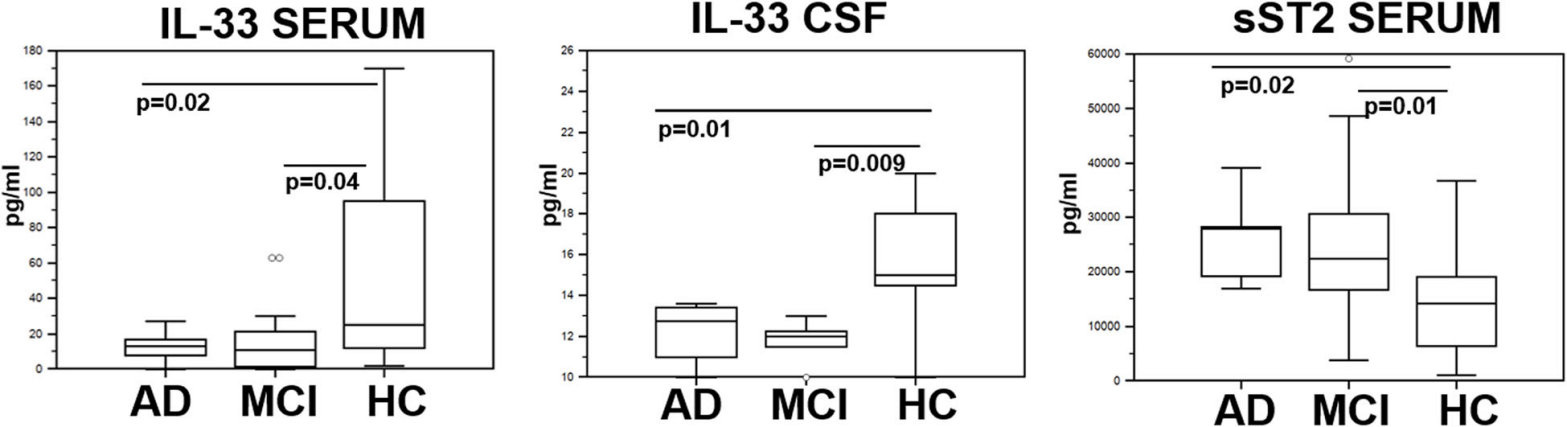
IL-33 and sST2. IL-33 and sST2 concentration in serum and CSF of AD, MCI, and age- and sex-matched HC individuals (*N* = 30 in each group) was analyzed by ELISA in undiluted samples. The boxes stretch from the 25° to the 75° percentile; the line across the boxes indicates the median values; the lines stretching from the boxes indicate extreme values. Statistical significance is shown

IL-33 concentration was decreased in serum and CSF of both AD and MCI individuals compared to HC (*p* value vs. HC: serum, AD *p* = 0.02; MCI *p* = 0.04; CSF, AD *p* = 0.01; MCI *p* = 0.009). In contrast with these results, serum concentration of the IL-33 decoy receptor sST2 was significantly increased in AD and MCI compared to HC (*p* value vs. HC: *p* = 0.02 and *p* = 0.01, respectively) (Fig. 2). CSF sST2 concentration was below the limit of detection of the assay (33 pg/ml) in all the analyzed samples (data not shown).

### IL-1β, IL-6, and IL-10 serum and CSF concentration

**Fig. 3:**
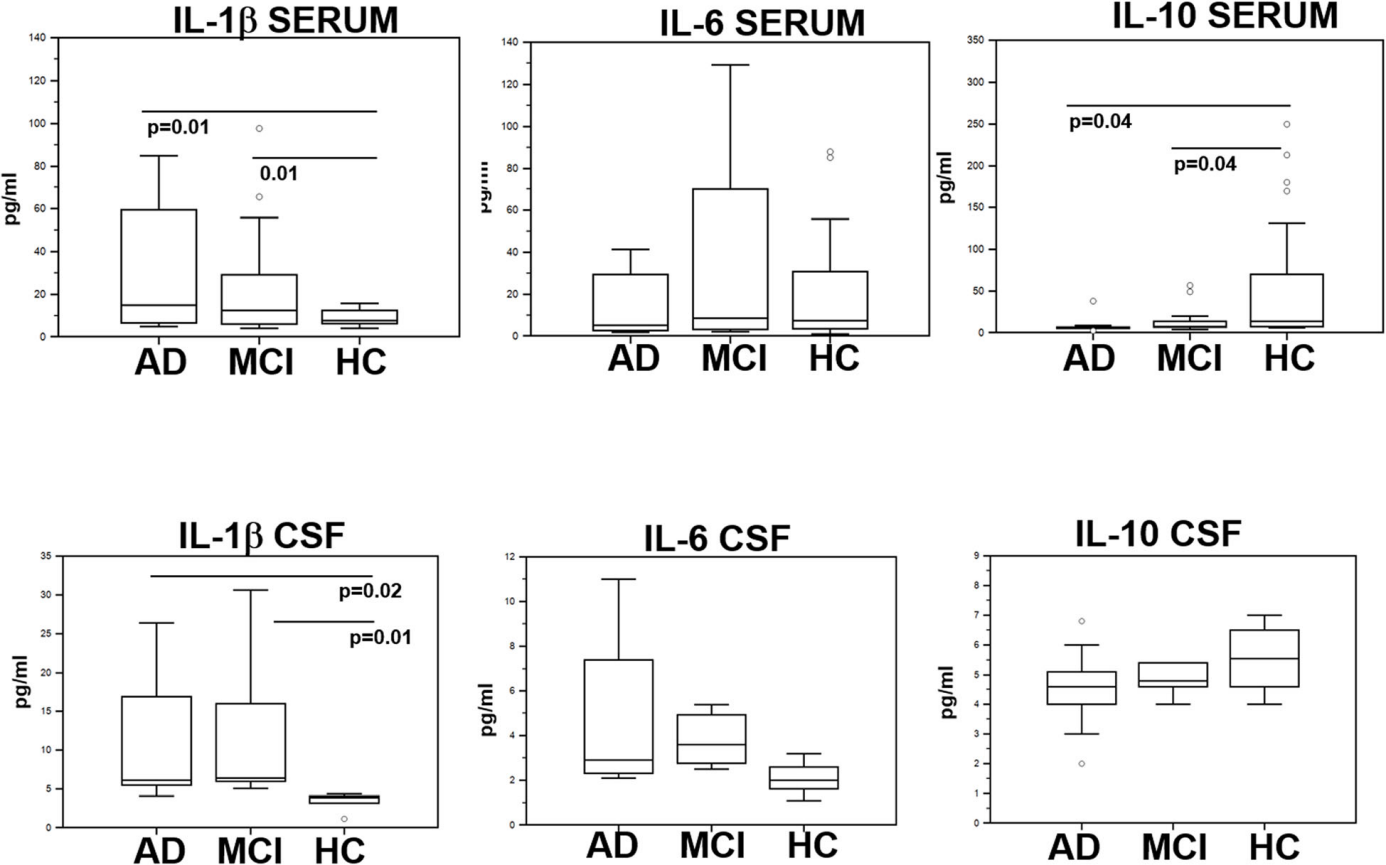
IL-1β, IL-6, and IL-10 concentration. IL-1β, IL-6, and IL-10 concentration in serum (upper panels) and CSF (lower panels) of AD, MCI, and age- and sex-matched HC individuals (*N* = 30 in each group). The boxes stretch from the 25° to the 75° percentile; the line across the boxes indicates the median values; the lines stretching from the boxes indicate extreme values. Statistical significance is shown

IL-1β serum concentration was significantly increased in AD and MCI sera compared to HC (*p* = 0.01 in both cases), whereas that of IL-10 was significantly reduced in both groups of patients compared to HC (*p* = 0.04 in both cases). No significant differences were detected when IL-6 serum concentration was compared between the three groups. These results are shown in Fig 3.

In CSF, IL-1β concentration was significantly augmented in AD and MCI compared to HC (*p* = 0.02 and *p* = 0.01, respectively). IL-6 concentration was slightly augmented and IL-10 concentration was marginally reduced in AD and MCI compared to what was seen in HC but these differences did not reach statistical significance (Fig. 3).

### Recombinant IL-33 differentially modulates IL-1β, IL-6, and IL-10 production by LPS-primed and Aβ_42_-stimulated PBMC

Monocytes of AD, MCI, and HC individuals (*n* = 8 in all cases) were LPS-primed and Aβ_42_-stimulated and IL-1β, IL-6, and IL-10 production was measured by ELISA. Whereas the production of these cytokines was minimal (< 20 pg/ml in all cases) and comparable between the three groups of individuals analyzed when monocytes were unstimulated (medium alone) or when IL-33 was added to the culture medium, LPS-primed, and Aβ_42_-stimulated monocytes of AD and MCI patients produce significantly augmented quantities of IL-1β (median, AD = 230 ng/ml; MCI = 140 ng/ml ) compared to HC (median, 62 ng/ml; AD vs. HC *p* = 0.006; MCI vs. HC *p* = 0.03). Notably, IL-10 production by cells stimulated in the same conditions was significantly reduced in AD (median, 129 ng/ml) compared to HC individuals (median, 245 ng/ml) (*p* = 0.01).

**Fig. 4:**
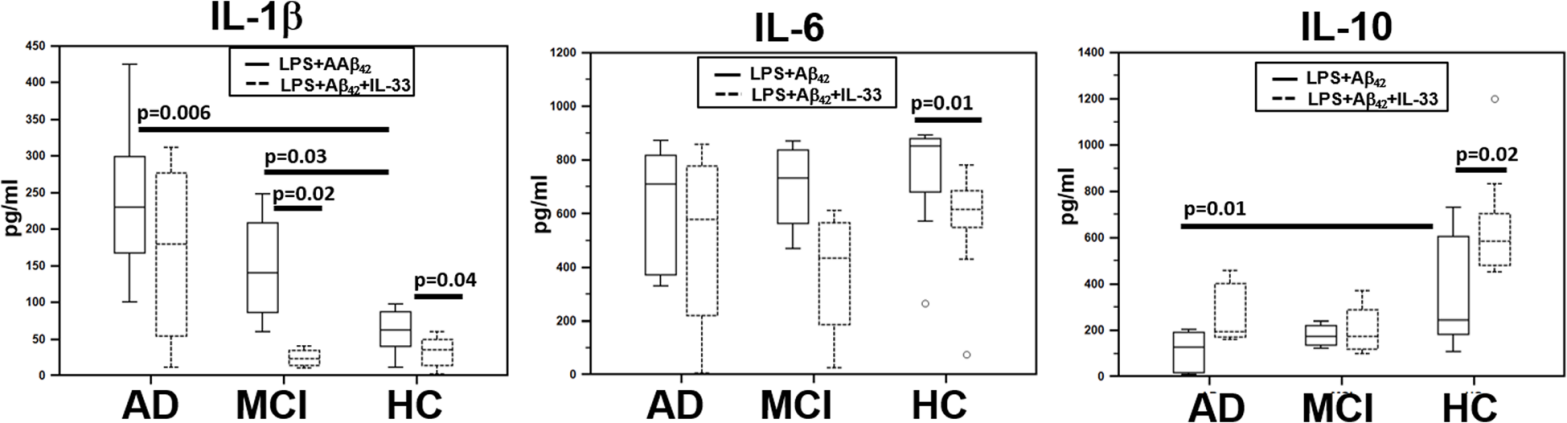
IL-1β, IL-6, and IL-10 production. IL-1β, IL-6, and IL-10 production by LPS-primed and Aβ_42_-stimulated monocytes of AD, MCI, and age- and sex-matched HC individuals (*N* = 8 in each group) in the absence (straight lines) or presence (dotted lines) of human recombinant IL-33 (10 ng/ml). The boxes stretch from the 25° to the 75° percentile; the line across the boxes indicates the median values

Addition of IL-33 to cell cultures resulted in a significant reduction of the production of both IL-1β (median, 36 pg/ml; *p* = 0.04) and IL-6 (median, 616 pg/ml; *p* = 0.01) in HC, and of IL-1β alone in MCI (median, 23 pg/ml; *p* = 0.02). Finally, LPS+Aβ_42_-stimulated IL-10 production was not modified by IL-33 in AD and MCI, but was significantly increased in HC (median, 585 pg/ml; *p* = 0.02) (Fig. 4).

These results support the idea that IL-33 reduces the generation of proinflammatory cytokines and stimulates that of anti-inflammatory cytokines, favoring the establishment of an anti-inflammatory milieu, and suggest that IL-10-mediated anti-inflammatory mechanisms are impaired/exhausted in AD and MCI.

### Recombinant IL-33 reduces NF-kB nuclear translocation

**Fig. 5:**
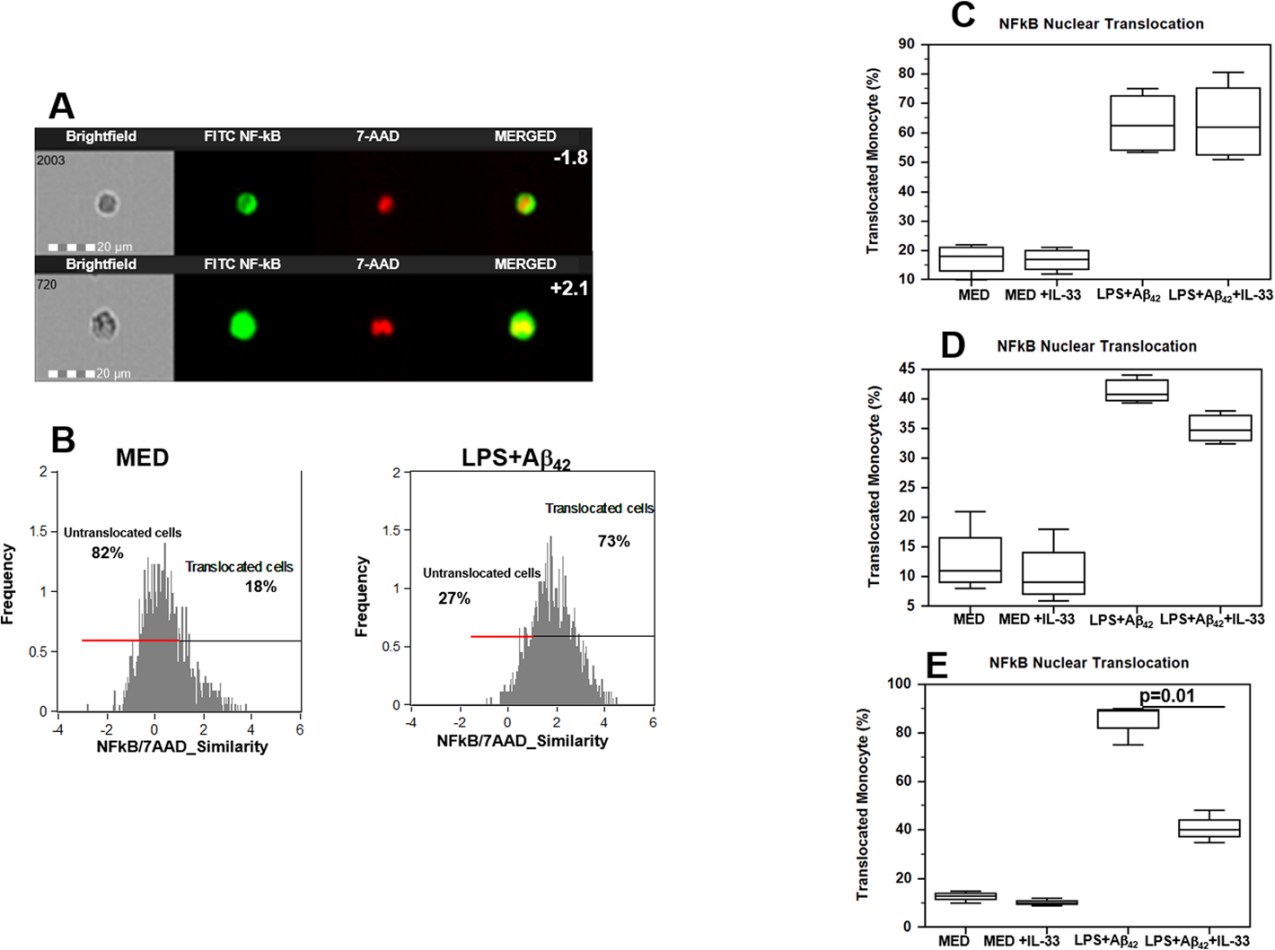
NF-kB nuclear translocation. **a** Representative images of NF-kB nuclear translocation in unstimulated (MED) and in LPS-primed and Aβ_42_ stimulated monocytes. First column = cells in brightfield (BF); second column = NF-kB-FITC fluorescence; third column = 7AAD fluorescence; fourth column = NF-kB and 7AAD merged fluorescence. Fourth column: the upper image showing the red nuclear image (7AAD staining) surrounded by the green cytoplasmic NF-kB staining indicates lack of nuclear translocation (similarity score = − 1.85); the lower image, where NF-kB and the nuclear dye are colocalized, indicates nuclear translocation (similarity score = + 2.3). **b** NF-κB/7-AAD similarity histograms indicating the percentage of cells in which NF-kB is untranslocated (negative similarity score) or translocated (positive similarity score) to the nucleus. **c**–**e** NF-kB nuclear translocation in unstimulated (MED) and in LPS-primed and Aβ_42_ stimulated monocytes in the absence or in the presence of human recombinant IL-33 (10 ng/ml); data obtained with cells of representative AD (**c**), MCI (**d**), and HC (**e**) individuals are shown. Summary results are presented in the bar graphs. The boxes stretch from the 25° to the 75° percentile; the line across the boxes indicates the median values. Statistical significance is shown

Unstimulated as well as LPS-primed and Aβ_42_-stimulated monocyte of AD (*n* = 5), MCI (*n* = 5), and HC individuals (*n* = 5) were analyzed to verify the effect of IL-33 on NF-kB nuclear translocation using the FlowSight technology. No differences in NF-kB nuclear translocation were detected in unstimulated cells (data not shown); upon antigenic stimulation, the IL-33 was observed to significantly reduce NF-kB nuclear translocation (*p* = 0.01) in HC alone (Fig. 5). These results suggest that this is a pivotal IL-33 anti-inflammatory mechanism that is possibly lost in MCI and AD.

## Discussion

Alzheimer’s disease is a highly prevalent form of dementia characterized by the accumulation of extracellular amyloid beta plaques in the brain, neuronal cell death, and neuroinflammation. The pathogenesis of AD-associated neuroinflammation includes an increased production of proinflammatory cytokines, possibly driven by the excessive activation of the inflammasome, a reduced activity of Treg lymphocytes, and the dysregulation of immune-mediated mechanisms of tolerance. Recent results suggest that IL-33 plays an important role as well in the pathogenesis of AD. IL-33 is released by damaged cell and can be produced by mast cells, macrophages, and dendritic cells either as an active, full length, or as an inactive differently cleaved form. Notably, although only present in a small minority of samples, the cleaved, biologically inert form of IL-33 was present in serum of a subset of AD patients alone.

IL-33 is endowed with both pro- and anti-inflammatory properties, hence the presence of contrasting data indicating both an increase and a reduction of IL-33 in AD. We investigated the role of IL-33 in AD pathogenesis both by directly measuring the concentration of this cytokine and its decoy receptor in biological fluids of AD and MCI patients and by analyzing the effect of IL-33 addition in an in vitro model of Aβ_42_-stimulated monocytes of patients and healthy controls.

Results herein show that the IL-33/ST2 axis is deeply impaired in MCI and AD. Thus, IL-33 concentration in serum and CSF was significantly reduced, whereas the concentration of sST2, the IL-33-specific decoy receptor, was significantly increased in the same patients. In AD, thus, lower amounts of IL-33 are produced and they cannot optimally bind their cognate receptor on the surface of cells, as they are trapped by soluble decoys.

That reduced concentrations of IL-33 are associated with inflammation was indirectly confirmed by the observation that IL1β concentrations were significantly increased whereas that of IL-10 was greatly reduced in the same AD and MCI patients. The anti-inflammatory role of IL-33 was further reinforced by the observation that IL-33 supplementation resulted in the reduction of IL-1β production by Aβ-stimulated cells of MCI and HC and of IL-6 by cells of HC alone. Importantly, IL-33 increased IL-10 production by cells of HC alone, suggesting that the ability of monocytes of AD and MCI individuals to secrete IL-10 in response to IL-33 is deeply defective or exhausted.

IL-1β is augmented in AD. This cytokine promotes amyloid plaque deposition, reduces phagocytic activity by the microglia, stimulates the hyperphosphorylation of τ protein, and affects synaptic plasticity. IL-10 production, on the other hand, has repeatedly been shown to be reduced in AD patients [24, 41, 42], in whom the IL-10 gene SNPs associated with higher production of this cytokine are also less frequently detected [33]. Results herein could thus at least partially justify the genesis of the proinflammatory mileu seen in AD and MCI as the consequence of the reduced amounts of IL-33 seen in these patients.

Notably, IL-33 significantly down-modulated NF-kB nuclear translocation in cells of HC alone, indicating this as possible mechanisms by which IL-33 favors the maintenance of an anti-inflammatory milieu in individuals in whom dementia is not present. Thus, IL-33 was shown to have a regulatory effect on the NF-kB pathway, which is mediated by its ability to binding the NF-kB p65 subunit [9]. The IL-33/NF-kB p65 complex impedes p65-mediated transactivation; this downregulates NF-kB activity, with a dampening effect on inflammation. The observation that this effect was lost in cells of MCI and AD individuals suggests that the role of IL-33 in the pathogenesis of AD- and MCI-associated inflammation is multifaceted, as this cytokine would exert its effect both at the intracellular and at the extracellular level. To summarize, (1) reduction of IL-33 production per se, (2) increased concentrations of the sST2 decoy receptor, and (3) an impairment of the ability of IL-33 to induce NF-kB nuclear translocation are different mechanism that can explain the proinflammatory role played by IL-33 in AD and MCI.

IL-33 was also reported to induce the polarization of monocytes toward an M2 anti-inflammatory phenotype; these monocytes produce IL-10, whose concentration, as indicated above, is greatly reduced in AD and MCI individuals [43, 44]. M2 polarization by IL-33 is possibly explained by the observation that the IL-33 binding to ST2 negatively regulates TLR signaling by competing with MyD88 [45, 46]. The IL-33/ST2 complex thus suppresses IL-1β generation and the down-stream activation of the TLR signaling pathway by sequestration of MyD88 with the consequence of further inhibiting NF-kB and activating MAP kinases [46]. IL-33 indeed activates ERK1\2 and STAT3, facilitating binding of STAT3 to the − 1954 to − 1936 bp sequence upstream of the IL-10 transcription start site, thereby promoting its transcription in macrophages [47]. Although we did not analyze M1 and M2 monocytes in this study, previously published data indicate that M2 cells are significantly reduced in AD. The reduction of M2 monocytes seen in AD could explain why IL-33 supplementation did not increase IL-10 production by monocytes of AD and MCI individuals in our in vitro system and could be a third way to justify why the lower quantities of IL-33 seen in AD results in inflammation in these patients.

IL-33 has repeatedly been suspected to be involved in the pathogenesis of CNS diseases. In particular, the administration of recombinant IL-33 was shown to promote recovery in a mouse model of autoimmune encephalomyelitis (EAE) [48, 49] and to provide neuroprotection in a mouse model of contusion spinal cord injury (SCI) [43]. In the APP/PS1 animal model of AD, IL-33 attenuated AD pathology and memory deficit and stimulated the polarization of microglia in an anti-inflammatory direction [34, 50]. Finally, recent data obtained in a small groups of MCI who did or did not convert to AD over time [31] showed that higher amounts of IL-33-producing CD14+ monocytes were seen in AD-non converters, in whom percentages of CD14+/IL-33+ cells positively correlated with the volumes of both left and right hippocampus.

Finally, recent data obtained in a small groups of MCI who did or did not convert to AD over time [31] showed that higher amounts of IL-33-producing CD14+ monocytes were seen in AD-non converters, in whom percentages of CD14+/IL-33+ cells positively correlated with the volumes of both left and right hippocampus. In the attempt to further analyze possible correlations between hippocampus volumes and IL-33, we are planning to verify the immunological effects of IL-33 on human microglia cell lines.

## Conclusions

IL-33 was shown to have a neuroprotective role in AD secondary to the reduction of Aβ secretion and the activation of Aβ phagocytosis by the microglia [35]. Findings herein offer an immune explanation as well to the protective role of IL-33 in AD; these results warrant the investigation of this cytokine in treatment and rehabilitation programs for AD.

## Data Availability

The authors confirm that the data supporting the findings of this study are available within the article. The raw data of this study are available from the corresponding author [M.S.] on request.

## Abbreviations

AD: Alzheimer’s disease
MCI: Mild cognitive impairment
HC: Healthy controls
IL: Interleukin
CSF: Cerebrospinal fluid
Aβ: Amyloid beta
TLR: Toll-like receptor
PBMC: Peripheral blood mononuclear cell
sST2: Soluble ST2

## References

[1] Li-Xia D, Yan-Qing W, Guo-Qiang H, Wen-Li M (2018). A IL-33/ST2 Pathway as a rational therapeutic target for CNS diseases. Neuroscience..

[2] Luthi AU, Cullen SP, McNeela EA, Duriez PJ, Afonina IS, Sheridan C (2009). Suppression of interleukin-33 bioactivity through proteolysis by apoptotic caspases. Immunity..

[3] Cayrol C, Girard JP (2009). The IL-1-like cytokine IL-33 is inactivated after maturation by caspase-1. Proc Natl Acad Sci USA..

[4] Lefrançais E, Roga S, Gautier V, Gonzalez-de-Peredo A, Monsarrat B, Girard JP (2012). IL-33 is processed into mature bioactive forms by neutrophil elastase and cathepsin G. Proc Natl Acad Sci USA..

[5] Lefrançais E, Duval A, Mirey E, Roga S, Espinosa E, Cayrol C (2014). Central domain of IL-33 is cleaved by mast cell proteases for potent activation of group-2 innate lymphoid cells. Proc Natl Acad Sc USA..

[6] Nikolas TM, Michael UM (2016). Interleukin 33 is a guardian of barriers and a local alarmin. Nature Immunology..

[7] Reverchon F, Mortaud S, Sivoyon M, Maillet I, Laugeray A, Palomo J (2017). IL-33 receptor ST2 regulates the cognitive impairments associated with experimental cerebral malaria. PLoS Pathog..

[8] Chackerian AA, Oldham ER, Murphy EE, Schmitz J, Pflanz S, Kastelein RA (2007). IL-1 receptor accessory protein and ST2 comprise the IL-33 receptor complex. J Immunol..

[9] Ali S, Mohs A, Thomas M, Klare J, Ross R, Schmitz ML (2011). The dual function cytokine IL-33 interacts with the transcription factor NF-kappaB to dampen NF-kappaB-stimulated gene transcription. J Immunol..

[10] Sanada S, Hakuno D, Higgins LJ, Schreiter ER, McKenzie AN, Lee RT (2007). IL-33 and ST2 comprise a critical biomechanically induced and cardioprotective signaling system. J Clin Inves..

[11] Peine M, Marek RM, Löhning M (2016). IL-33 in T cell differentiation, function, and immune homeostasis. Trends Immunol..

[12] Halim TYF, Krauss RH, Sun AC, Takei F (2012). Lung natural helper cells are a critical source of Th2 cell-type cytokines in protease allergen-induced airway inflammation. Immunity..

[13] Besnard AG, Togbe D, Guillou N, Erard F, Quesniaux V, Ryffel B (2011). IL-33-activated dendritic cells are critical for allergic airway inflammation. Eur. J. Immunol..

[14] Halim TYF, Steer CA, Mathä L, Gold MJ, Martinez-Gonzalez I, McNagny KM (2014). Group 2 innate lymphoid cells are critical for the initiation of adaptive T helper 2 cell-mediated allergic lung inflammation. Immunity..

[15] Kurowska-Stolarska M, Stolarski B, Kewin P, Murphy G, Corrigan CJ, Ying S (2009). IL-33 amplifies the polarization of alternatively activated macrophages that contribute to airway inflammation. J.Immunol..

[16] Kondo Y, Yoshimoto T, Yasuda K, Futatsugi-Yumikura S, Morimoto M, Hayashi N (2008). Administration of IL-33 induces airway hyper responsiveness and goblet cell hyperplasia in the lungs in the absence of adaptive immune system. Int Immunol..

[17] Schiering C, Krausgruber T, Chomka A, Fröhlich A, Adelmann K, Wohlfert EA (2014). The alarmin IL-33 promotes regulatory T-cell function in the intestine. Nature.

[18] Molofsky AB, Van Gool F, Liang HE, Van Dyken SJ, Nussbaum JC, Lee J (2015). Interleukin-33 and Interferon-c counter-regulate group 2 innate lymphoid cell activation during immune perturbation. Immunity..

[19] Nascimento DC, Melo PH, Piñeros AR, Ferreira RG, Colón DF, Donate PB (2017). IL-33 contributes to sepsis-induced long-term immunosuppression by expanding the regulatory T cell population. Nat Commun..

[20] Marsh SE, Abud EM, Lakatos A, Karimzadeh A, Yeung ST, Davtyan H (2016). The adaptive immune system restrains Alzheimer’s disease pathogenesis by modulating microglial function. Proc Natl Acad Sci USA..

[21] Zhu D, Yang N, Liu YY, Zheng J, Ji C, Zuo PP (2016). M2 macrophage transplantation ameliorates cognitive dysfunction in amyloid-beta-treated rats through regulation of microglial polarization. J Alzheimer’s Dis..

[22] Dansokho C, Ait Ahmed D, Aid S, Toly-Ndour C, Chaigneau T, Calle V (2016). Regulatory T cells delay disease progression in Alzheimer-like pathology. Brain..

[23] Saresella M, Calabrese E, Marventano I, Piancone F, Gatti A, Calvo MG (2010). PD1 negative and PD1 positive CD4+ T regulatory cells in mild cognitive impairment and Alzheimer’s disease. J Alzheimers Dis..

[24] Saresella M, Calabrese E, Marventano I, Piancone F, Gatti A, Farina E, et al. A potential role for the PD1/PD-L1 pathway in the neuroinflammation of Alzheimer’s disease. Neurobiol Aging. 2012;33:624.e11-22. 10.1016/j.neurobiolaging.2011.03.004 21514692

[25] Saresella M, La Rosa F, Piancone F, Zoppis M, Marventano I, Calabrese E (2016). The NLRP3 and NLRP1 inflammasomes are activated in Alzheimer’s disease. Mol Neurodegener..

[26] Halle A, Hornung V, Petzold GC, Stewart CR, Monks BG, Reinheckel T (2008). The NALP3 inflammasome is involved in the innate immune response to amyloid-beta. Nat Immunol..

[27] Heneka MT, Kummer MP, Stutz A, Delekate A, Schwartz S, Vieira-Saecker A (2013). NLRP3 is activated in Alzheimer’s disease and contributes to pathology in APP/PS1 mice. Nature..

[28] McKhann GM, Knopman DS, Chertkow H, Hyman BT, Jack CR, Kawas CH (2011). The diagnosis of dementia due to Alzheimer’s disease: recommendations from the National Institute on Aging-Alzheimer’s Association workgroups on diagnostic guidelines for Alzheimer’s disease. Alzheimers Dement..

[29] Petersen RC, Smith GE, Waring SC, Ivnik RJ, Tangalos EG, Kokmen E (1999). Mild cognitive impairment. Arch Neurol..

[30] Baglio F, Saresella M, Preti MG, Cabinio M, Griffanti L, Marventano I (2013). Neuroinflammation and brain functional disconnection in Alzheimer’s disease. Front Aging Neurosci..

[31] La Rosa F, Saresella M, Baglio F, Piancone F, Marventano I, Calabrese E (2017). Immune and imaging correlates of mild cognitive impairment conversion to Alzheimer’s disease. Sci Rep..

[32] Cabinio M, Saresella M, Piancone F, LaRosa F, Marventano I, Guerini F (2018). Association between hippocampal shape, neuroinflammation, and cognitive decline in Alzheimer’s disease. J Alzheimers Dis..

[33] Arosio B, Trabattoni D, Galimberti L, Bucciarelli P, Fasano F, Calabresi C (2004). Interleukin-10 and interleukin-6 gene polymorphisms as risk factors for Alzheimer’s disease. Neurobiol Aging..

[34] Fu AK, Hung KW, Yuen MY, Zhou X, Mak DS, Chan IC (2016). IL-33 ameliorates Alzheimer’s disease-like pathology and cognitive decline. Proc Natl Acad Sci U S A..

[35] Chapuis J, Hot D, Hansmannel F, Kerdraon O, Ferreira S, Hubans C (2009). Transcriptomic and genetic studies identify IL-33 as a candidate gene for Alzheimer’s disease. Mol Psychiatry..

[36] Xiong Z, Thangavel R, Kempuraj D, Yang E, Zaheer S, Zaheer A (2014). Alzheimer’s disease: evidence for the expression of interleukin-33 and its receptor ST2 in the brain. Journal of Alzheimer’s Disease..

[37] Dubois B, Feldman HH, Jacova C, Hampel H, Molinuevo JL, Blennow K, et al. Advancing research diagnostic criteria for Alzheimer’s disease: the IWG-2 criteria. Lancet Neurol. 2014 Jun;13(6):614-29. doi: 10.1016/S1474-4422(14)70090-0. Erratum in: Lancet Neurol. 2014 Aug;13(8):757. 10.1016/S1474-4422(14)70090-0 24849862

[38] Folstein MF, Folstein SE, McHugh PR (1975). Minimental state. A practical method for grading the cognitive state of patients for the clinician. J Psychiatr Res..

[39] Hughes CP, Berg L, Danziger WL, Coben LA, Martin RL (1982). A new clinical scale for the staging of dementia. Br J Psychiatry..

[40] Ligthart GJ, Corberand JX, Fournier C, Galanaud P, Hijmans W, Kennes B (1984). Admission criteria for immunogerontological studies in man: the SENIEUR protocol. Mech Ageing Dev..

[41] Saresella M, Calabrese E, Marventano I, Piancone F, Gatti A, Alberoni M, Nemni R, Clerici M (2011). Increased activity of Th-17 and Th-9 lymphocytes and a skewing of the post-thymic differentiation pathway are seen in Alzheimer’s disease. Brain Behav Immun..

[42] Saresella M, Marventano I, Calabrese E, Piancone F, Rainone V, Gatti A, Alberoni M, Nemni R, Clerici M (2014). A complex proinflammatory role for peripheral monocytes in Alzheimer’s disease. J Alzheimer’s Dis..

[43] Pomeshchik Y, Kidin I, Korhonen P, Savchenko E, Jaronen M, Lehtonen S (2015). Interleukin-33 treatment reduces secondary injury and improves functional recovery after contusion spinal cord injury. Brain Behav Immun..

[44] Schmitz J, Owyang A, Oldham E, Song Y, Murphy E, McClanahan TK (2005). IL-33, an interleukin-1-like cytokine that signals via the IL-1 receptor-related protein ST2 and induces T helper type 2-associated cytokines. Immunity..

[45] Liu J, Buckley JM, Redmond HP, Wang JH (2010). ST2 negatively regulates TLR2 signaling, but is not required for bacterial lipoprotein-induced tolerance. J Immunol..

[46] Brint EK, Xu D, Liu H, Dunne A, McKenzie AN, O’Neill LA (2004). ST2 is an inhibitor of interleukin 1 receptor and Toll-like receptor 4 signaling and maintains endotoxin tolerance. Nat Immunol..

[47] Zhang HF, Wu MX, Lin YQ, Xie SL, Huang TC, Liu PM (2017). IL-33 promotes IL-10 production in macrophages: a role for IL-33 in macrophage foam cell formation. Exp Mol Med..

[48] Jiang HR, Milovanović M, Allan D, Niedbala W, Besnard AG, Fukada SY (2012). IL-33 attenuates EAE by suppressing IL-17 and IFN-γ production and inducing alternatively activated macrophages. Eur J Immunol..

[49] Milovanovic M, Volarevic V, Ljujic B, Radosavljevic G, Jovanovic I, Arsenijevic N (2012). Deletion of IL-33R (ST2) abrogates resistance to EAE in BALB/C mice by enhancing polarization of APC to inflammatory phenotype. PLoS One..

[50] Zhu D, Yang N, Liu YY, Zheng J, Ji C, Zuo PP (2016). M2 macrophage transplantation ameliorates cognitive dysfunction in amyloid-beta-treated rats through regulation of microglial polarization. JAlzheimer’s Dis..

